# Supplementary material for: Advanced informatics understanding of clinician-patient communication: A mixed-method approach to oral health literacy talk in interpreter-mediated pediatric dentistry
Source: PLoS One. 2020 Mar 20;15(3):e0230575. doi: 10.1371/journal.pone.0230575 (PMC7083275; doi:10.1371/journal.pone.0230575)
Supplement: S1 File — (DOCX) [file pone.0230575.s001.docx]

## S1 Appendix 1: Transcription conventions with Jeffersonian notation

D: Dentist;

DSA: Dental surgery assistant;

P: Parent/ Primary caregiver;

SD: Senior dentist

**Intonation marks:**

.: falling intonation

,: level intonation

! ? [: rising intonation

[: the beginning of overlapping

]: the end of overlapping

A:: XXX? B: ¼XXX B's turn is latched onto A's

:: lengthened sound (more colons mean greater length)

XXX: stressed words

(( )): non-verbal features or transcriber's comment

(.): noticeable pause shorter than 0.5 s (including regular pauses between sentences)

(~n): timed pause (in approximation) where “n” indicates the interval measured in

seconds
