# Supplementary material for: Advanced informatics understanding of clinician-patient communication: A mixed-method approach to oral health literacy talk in interpreter-mediated pediatric dentistry
Source: PLoS One. 2020 Mar 20;15(3):e0230575. doi: 10.1371/journal.pone.0230575 (PMC7083275; doi:10.1371/journal.pone.0230575)
Supplement: S2 File — (DOCX) [file pone.0230575.s002.docx]

## S2 File. Transcripts and patient information

Excerpt 1, Consultation 3 – “The patient’s tooth brushing behaviors”

Patient information: An eight-year-old girl is here for follow-up and a possible accident causing mal-alignment of teeth.

| Turn | Speaker | Conversation |
| --- | --- | --- |
| 73 | D | (.) okay::: (.) so:: Is she:: Are you (.) are you? Is she brushing:: (.) [the::?] |
| 74 | DSA | ehh::家長]係唔係:: 你幫小朋友 刷刷牙架？  [ehh::parent]Have:: you helped little buddy ((here to)) ((your daughter to)) brush teeth gah ? |
| 75 | P | [ehh:: (.) 如果夜]晚 有喺度就::會:: 望住佢:: 刷刷囉:: 因為 買咗隻電動::牙刷刷刷::畀佢自己刷刷::=  [ehh:: (.) If night]time I am here then::will:: observe her:: brushing loh:: because bought the electric::toothbrush::for her to brush it herself::= |
| 76 | D | [or:: she brushes on her own] |
| 77 | DSA | =ohh::: (.) mommy buy the electric brush to the:: [patient her] |
| 78 | D | [the child::] |
| 79 | DSA | self to brush |
| 80 | D | (.) al:right:: uhh::: electric toothbrush is okay:: she can [use it::] |
| 81 | DSA | [電動牙刷刷冇:]問題嘅=  [electric toothbrush no:]problem geh= |
| 82 | D | =but:= |
| 83 | DSA | =huh= |
| 84 | D | =but: you may have:: to help: [be:cause:::] |
| 85 | DSA | [但係你::呢]最好幫一幫佢:  [but you::leh]better help her: a bit |
| 86 | P | emm [emm] |
| 87 | DSA | [擦牙]  [ (.) toothbrushing] |

Excerpt 2, Consultation 3 – “Suggestion for daily tooth brushing”

Patient information: An eight-year-old girl is here for follow-up and a possible accident causing mal-alignment of teeth.

| Turn | Speaker | Conversation |
| --- | --- | --- |
| 130 | D | (.) and even:: here is almost out. Need (to) brush inner ::= |
| 131 | DSA | e  =This:: one::here is almost::out [yes::lah:]gem::Hence::change ehh:to brush a bit inner ((backward)) when brushing= |
| 132 | P | [Hmm:] |
| 133 | P | =刷刷入尐:=  =brush inner a bit:= |
| 134 | DSA | =係::l啦:: 你幫::佢::刷刷:::=  =Yes::lah:: you help::her::brush:::= |
| 135 | P | =H[mm:] |
| 136 | DSA | [佢::]自己刷刷咗先呢:: 跟住你::: 幫佢補:::=  [She::]herself brushes first leh:: then you::: help her brush::: ((again))= |
| 137 | DSA | =刷刷[啊::]好唔好ahh::? 因為佢::=  =brush[ahh::] Okay or not ahh::? Because she::= |
| 138 | P | [Hmm:] |
| 139 | DSA | =[始終都係]好::細::個::=  =[ ((she)) is still]very::small::((still a kid))= |
| 140 | D | [Al::right?] |
| 141 | P | =Hmm:= |
| 142 | DSA | =咁::你幫:::佢::刷刷(.)乾淨尐啊::[ (.) ] huh::佢都係未必:做得到:  =Then::you help:::her::brush (.) ((make it)) cleaner a bit ahh::[ (.) ] huh::she ((now)) may not be able: ((to)) do that well: ((yet)) |
| 143 | P | [好啊::]  [okay ahh::] |
| 144 | DSA | 同埋你都::見到好多牙垢:膜 刷刷牙嘅時候刷 刷埋尐牙肉邊::: ((mom nodding)) 最容易蛀嘅:::嗰尐位[置]=  Also you can::see a lot of plaque. When brushing brush the gum sides::: ((mom nodding)) get caries most easily get:::those si[tes]= |
| 145 | P | [Hmm:] |

Excerpt 3, Consultation 1 – “Dental caries prevention work after meals”

Patient information: An eight-year-old boy previously treated under general anesthesia has erupting permanent teeth.

| Turn | Speaker | Conversation |
| --- | --- | --- |
| 215 | D | =See if it's his breakfast (.) he goes: to school (.) there's food: in:side (.) then:: (.) ahh:: (.) there's very high chance of caries= |
| 216 | DSA | =ahh:: (.) 嗱因為呢:: (.) 佢:大人牙出咗啦::=  =ahh:: (.) here because leh::(.)his: permanent teeth already erupted lah::= |
| 217 | P | =Hmm::= |
| 218 | DSA | =咁:: (.) ehh:: (.) 其實呢度 (.) 呢上邊四隻 (.)下邊四隻呢:: (.) 比較容易刷尐架啦 (.) 咁::如果尐食物°容易°黐住喺牙罅中間: (.) 佢又唔飲水 (.) 又唔會刷牙架話::=  =then:: (.) ehh:: (.) actually here (.) leh four ((teeth)) above (.) four teeth below leh:: (.) are relatively easy to brush (.) Then::if the food easily got stuck over space between teeth: (.) he then do not drink water (.) and ((if)) do not brush teeth::= |
| 219 | DSA | =[返咗學呢:]  =[after going to school leh:] |
| 220 | P | [飲(.)食完有飲]  [drink (.) did drink after eating] |
| 221 | DSA | (.) 係啊:: (.) 係囉:: (.) 咁 (.) 即係沖唔去囉:: (.) 咁啊 (.) 最好用尐: (.) ehh::: (.) 牙籤啊:: (.) 尐牙線啊:: (.) 幫佢清潔囉:: (.) 即係如果佢食完嘢:: (.)你冇:理佢:: (.)佢返咗學嘅話呢:: (.) 佢好::高: (.) 嘅成分呢: (.) 會蛀牙嘅=  (.) yes ahh:: (.) yes loh:: (.) then (.) that is cannot flush away loh:: (.) then ahh (.) best ((way is)) to use a: (.) ehh::: (.) tooth pick ahh:: (.) the dental floss ahh:: (.) help him clean up loh:: (.) that is if he finishes eating:: (.) ((if)) you do not: ((take)) care ((of)) him:: (.) if he ((then)) goes to school leh:: (.) he very high::: (.) chance leh: (.) will cause caries = |
| 222 | P | =ohh(.)好啊 ((nodding))  =ohh(.)okay ahh ((nodding)) |
| 223 | DSA | (.) 因為太:耐呢[:: (.) 積住]呢 (.) 喺個牙罅中間  (.) because ((after a)) long:time leh[:: (.) ((if)) got stuck]leh (.) over the middle of the gaps between teeth |
| 224 | P | [係啊 (.) 我知:]  [yes ahh (.) I know:] |
| 225 | DSA | 位: (.) 尐牙面嘅[話呢::]  spot: (.) ((over)) those tooth surface [leh::] |
| 226 | P | [Hmm::] |
| 227 | DSA | (.) 因為你都:見到:佢隻門牙嚟嘅啫喎::(.)he ((laughing a bit)) (.)都: (.) 咁::[多]  (.) because as you:also see: ((it’s)) just his front teeth ((here))only wor:: (.) he ((laughing a bit)) (.) already: (.) so::[many] ((caries)) |

Excerpt 4, Consultation 11 – “Inquiring about the patient’s daily tooth brushing behaviors”

Patient information: A seven-year-old boy is here for his six-month review after previous treatment under general anesthesia.

| Turn | Speaker | Conversation |
| --- | --- | --- |
| 34 | D | [if:]She is:: helping:: the child:: to brush?= |
| 35 | DSA | =emm::= |
| 36 | D | =ahh:: what (.) kind of toothpaste:: they use?::= |
| 37 | DSA | =emm[::] |
| 38 | D | [how:]: many times do they brush the teeth?:= |
| 39 | DSA | =ahh:小朋友宜家呢::一日刷幾多次牙啊?=  =ahh: ((your child)) now::how many times ((he)) brushed ((his)) teeth per day?= |
| 40 | P | =ehh: 兩次  =ehh: twice |
| 41 | DSA | brush twice daily [用乜]嘢牙膏啊?  brush twice daily [using which] ((type of)) toothpaste? |
| 42 | D | [ok:] |
| 43 | P | eh[h:: 都係]  eh[h:: ((it’s)) also] |
| 44 | DSA | [乜嘢牌子]啊?  [which brand]ahh? |
| 45 | DSA | 有冇含有氟素嘅呢？=  ((does it)) have any fluoride ((in it))？= |
| 46 | P | =有啊有啊有啊=  =yes yes it has= |
| 47 | DSA | =邊個牌子啊? [Oral B ahh: Colgate:] ahh::  =Which brand is it ahh? [Oral B ahh: Colgate:] ahh:: |
| 48 | P | [我都係買::]  [I always buy::] |
| 49 | P | Oral B ahh:係啊係啊=  Oral B ahh:yes ahh yes ahh= |
| 50 | DSA | =use the Oral B:: uhh:: too[thpaste:] |
| 51 | P | [有時都:]會買::獅王嗰隻=  [at times ((I)) will:]buy::the Lion that ((brand))= |
| 52 | DSA | =sometimes use the:Lion:: |
| 53 | D | al:right:: |
| 54 | DSA | brand: |
| 55 | D | (.) so I think that: has:: (.) fluoride::= |
| 56 | DSA | =have fluoride:= |
| 57 | D | =ya: |
| 58 | DSA | (.) 咁::宜家一日刷:兩次你::有:冇:: 幫小朋[友刷定係]佢自己刷?=  (.) so::now brushing: twice a day have::you:: helped[ him brush or]he does the brushing?= |
| 59 | P | [有我都有]  [yes I do ((help him brush his teeth)) ] |
| 60 | P | =都有[幫佢°刷°]  = ((yes I did)) [helped him°brush°] |
| 61 | DSA | [佢自己刷咗先]你:再幫佢補刷:?=  [he first does the brushing] you:then help him out:?= |
| 62 | P | =係啦係啦=  =yes lah yes lah= |
| 63 | DSA | =ok: ((to the dentist)) the patient will brush himself first: (.) and then:: af:ter:: mommy will help the patient to brush: tooth again: |
| 64 | D | o::k: |
| 65 | DSA | 兩次都有幫手hor:?  The twice ((daily)) brushing ((you)) help out hor:? |
| 66 | P | 我都::有啊  ((yes)) I::do |

Excerpt 5, Consultation 2 – “Giving instructions for the pulpectomy treatment”

Patient information: A seven-year-old boy has missing permanent teeth.

| Turn | Speaker | Conversation |
| --- | --- | --- |
| 18 | DSA | =頭先刷咗:膏膏啦: (.) 咁: (.) 跟住我哋滴尐神奇藥水:: (.) 令到隻牙仔瞓覺豬(.)咁:(.)跟住呢:(.)我哋(.)記唔記得(.)要落一個: (.)  =just now applied:some cream cream ((Cantonese child lay term for cream)) lah: (.) then: (.) later we drop a bit magic medical potion ((solution)):: (.) making the tooth fall asleep (.) then: (.) later leh: (.) we (.) remember (.) need to lay one: (.) |
| 19 | SD | [can we] do it quick? |
| 20 | DSA | [uhh::] |
| 21 | D | you mean: (.) like (.) uhh:[af::ter this appointment but then (.) that will be bi:lateral (.) like (.) inject:ion]= |
| 22 | DSA | [呢尐: (.)一個戒指:: (.) 戴落隻牙仔度]  [this: (.) one ring:: (.) lay onto the tooth] |
| 23 | SD | =[because I can (.) I: (.) no no not (.) uhh::]= |
| 24 | DSA | [咁跟住呢: (.) 就放一個:]  [then next thing leh: (.) will put one:] |
| 25 | D | =[ohh cha (.) you mean like:: within a week]= |
| 26 | DSA | [咁樣嘅雨:褸:: (.) 咁: (.) 尐]=  [((a)) rain:coat::like that (.) then: (.) the]= |
| 27 | SD | [ (.) ya because]= |
| 28 | SD | =[I can see:: the:::]= |
| 29 | DSA | [水水呢::喺口裡面嗰尐呢:]  [water water ((lay term for saliva)) leh::those inside mouth leh:] |
| 30 | SD | =[the canine: can: (.) move (.) distally] |
| 31 | DSA | [姐姐幫你吸嗮:去:: (.) 咁你試下擘大個口:]  [sister ((means I)) ((performs)) suction for you: ((cleaning all your saliva)) (.) then you try open wide your mouth:] |

Excerpt 6, Consultation 6 – “Giving general suggestions to the patient”

Patient information: A seven-year-old-girl is brought in as she has caries.

| Turn | Speaker | Conversation |
| --- | --- | --- |
| 127 | DSA | 嗱我哋瞓[高小小先妹妹: 瞓:高尐]係::lah 呢度:  Now we lie[higher a bit first dear: lie:higher]yes::lah here: |
| 128 | D | [Al:right:: just:] |
| 129 | D | mir:ror[::] |
| e | DSA | [係::] lah:我哋有個::鏡鏡::睇下尐牙仔先:::係啦擘:大:大:=  [Yes::] lah:we have a::little mirror:: ((to)) check those little teeth first:::yes lah open:wi:der:= |
| 131 | D | e |
| 132 | DSA | =It's broken wor: |
| 133 | D | wor:::= |
| 134 | DSA | =huh. Ok::continue lah |
| 135 | D | He:((laugh a bit)) |
| 136 |  | ((silence)) |
| 137 | D | Yea::all is: al:right:: the sixes are al:most::out:: is no::caries:: I think we can dis:charge: her::= |
| 138 | DSA | =o::k |
| 139 | D | And she is not [well:: Oh yeh:: she's not well] |
| 140 | DSA | [好好喎::: (.) 媽媽幫佢刷刷得好乾淨]喎::huh:: ((Doctor leaving the bay))  [very good wor::: (.) mommy helps her brush cleanly]wor::huh:: ((Doctor leaving the bay)) |
| 141 | DSA | 記住啦喎::一日刷刷兩次牙仔:: 知唔知:啊:::? ((kid's nodding)) Hmm朝又早起身刷刷一次啦::臨瞓前將佢刷刷多一次跟住先瞓覺 °平時°唔好食甜嘢喎::因為你知啦::佢係全身麻醉做::補牙啊:::牙套咁樣呢: 我::哋都唔想再::幫佢做:全身麻醉尐嘢啦::=  Remember lah wor::two tooth brushing per day:: know or not:ahh:::? ((kid's nodding)) Hmm early morning after waking up brush once lah:: Before going to bed another ((teeth)) brushing. Only then ((you)) sleep. °On ((most)) of the days° don’t take in sweet food wor::because you already knew lah:: She had the GA ((general anesthesia)) for::teeth filling ahh:::adding a crown so forth leh: We don’t want to re::do:those things under GA lah::= |
| 142 | P | =係啊 [係啊]  =Yes ahh [yes ahh] |
| 143 | DSA | [刷刷得乾]淨咁一路咁樣換:牙::咁都ok:  [Brush]clean then all the way ((with)) change: ((of)) tooth:: Then also ok: |
| 144 | DSA | [啊嘛?]  [Right?] |
| 145 | P | [都ok:]ahh:ohh:  [All ok:]ahh:ohh: |
| 146 | DSA | 咁佢又參加咗學童保健啦: [係唔係?]咁::佢照:跟返::學童保健嘅度繼續返去做補牙或者有有需要呢: 就做:ehh::洗牙咁麼樣::=  Then she also participated in SDCS lah: [right?] Then:: she just:stick:: ((with)) SDCS ((program)) there. Continue ((the)) teeth filling or if necessary leh: Then get:ehh:: scaling so forth::= |
