# Supplementary material for: Advanced informatics understanding of clinician-patient communication: A mixed-method approach to oral health literacy talk in interpreter-mediated pediatric dentistry
Source: PLoS One. 2020 Mar 20;15(3):e0230575. doi: 10.1371/journal.pone.0230575 (PMC7083275; doi:10.1371/journal.pone.0230575)
Supplement: Attachment — Submitted filename: PONE-D-19-27918.docx [file pone.0230575.s003.docx]

PONE-D-19-27918

**General**

This is an interesting topic and it is good to see Discursis being applied to dentistry. The nature of the study, using children with their parents is also novel. I really like this paper and think it will be a valuable contribution to the literature but, as my comments below suggest, another revision is needed.

**Introduction**

The introduction was appropriate and well referenced. It set the scene with clear research questions that were relevant to the study. There were some awkward turns of phrase that need to be addressed.

Method

Again, the method section is clear. I think it might be useful to place the communication strategies section in the introduction as it builds on the information provided and does not relate to any methodology and data collection. I also think participant information should be in the Method section. In addition, I did not see a full procedure section beyond the little section in 2.1. I really want to see more information that outlines how the study was conducted with respect to recruitment and ages of children, education levels of parents etc. More is required here in order to better understand results.

**Results**

If the results section commences at 3.2, then I think more information is required about the three principle dimensions and how they were derived. I am unclear how you decided on the actual plots. Given we know nothing about the participants, it does not assist in understanding the generalizability of these findings.

It is always difficult to explain Discursis and the meaning of the diagonals in terms of engagement and time. Too much makes for a complex and unwieldy explanation. Too little leaves the reader unclear. The secret is to strike a balance. I do no think you are there yet. Some earlier explanation about how Discursis works would assist. At the moment I think you assume too much knowledge on the part of the reader. As someone who can understand these plots, I understand what you are saying but with the lack of information about participants and the lack of detail on Discursis, I think I would be in the minority. As an example, I believe you need to introduce the idea of recurrence patterns before the Results section (Line 182).

**Discussion**

I returned to lines 106 to 113 and felt that these aims had not been clearly linked with the results and subsequent discussion. You talk about ‘patient participation’ but remember it is parent participation in this instance. The importance of the mixed methodology was not emphasized sufficiently. Can the use of CA and Discursis be expanded?

**Minor**

Line 83: Hong Kong is an Asia's world city with a linguistic (should be Asian)

Line 88: Multilingual interpreters are essential resource (needs ‘an’)

Line 94: …. Challenges are existed (needs changing)

Line 110: The study also targeted to provide (needs changing)
