# Supplementary material for: Advanced informatics understanding of clinician-patient communication: A mixed-method approach to oral health literacy talk in interpreter-mediated pediatric dentistry
Source: PLoS One. 2020 Mar 20;15(3):e0230575. doi: 10.1371/journal.pone.0230575 (PMC7083275; doi:10.1371/journal.pone.0230575)
Supplement: Attachment — Submitted filename: Response to Reviewers_G.docx [file pone.0230575.s004.docx]

Dear Prof Barbara Schouten, Academic Editor,

Thank you for considering our research article entitled “Advanced informatics understanding of clinician-patient communication: a mixed-method approach to oral health literacy talks in interpreter-mediated pediatric dentistry” (PONE-D-19-27918). Enclosed please find a marked-up copy of our manuscript that highlights changes made to the original version, and an unmarked version of our revised paper without tracked changes. With regard to the Academic Editor and Reviewers’ comments, we provide the following point by point response and explanation.

**Comments from the Academic Editor:**

1. **Please ensure that your manuscript meets PLOS ONE's style requirements, including those for file naming. The PLOS ONE style templates can be found at** [**http://www.journals.plos.org/plosone/s/file?id=wjVg/PLOSOne_formatting_sample_main_body.pdf**](http://www.journals.plos.org/plosone/s/file?id=wjVg/PLOSOne_formatting_sample_main_body.pdf) **and** [**http://www.journals.plos.org/plosone/s/file?id=ba62/PLOSOne_formatting_sample_title_authors_affiliations.pdf**](http://www.journals.plos.org/plosone/s/file?id=ba62/PLOSOne_formatting_sample_title_authors_affiliations.pdf)

Response: We thank the Editor for the advice. We have revised the manuscript to meet PLOS ONS’s style as follows.

a. The information about the authors’ ZIP or Postal Codes, street addresses, or building/office numbers in the affiliations has been removed.
b. The symbols about authors’ contributorship have been added.
c. The format of the level 1 headings has been revised to be Bold type, 18pt front.
d. The format of figure citations has been revised from “Fig. X” (Figs. Y-Z) to “Fig X” (Figs Y-Z).
e. The format of figure titles has been revised to be Bold type.
f. The formats of the level 2 headings and the level 3 headings have been revised to be Bold type, 16pt front and Bold type, 14pt front, respectively.
g. The format of the references with more than six authors has been revised.

2. **Please provide further details in order to justify why only 77/199 of the larger dataset were included.**

Response: We thank the Editor for the concern about the selection of the 77 transcribed videos from a larger video-recorded corpus (n= 199). We have further explained the selection of the videos in the section “Material and methods - Data collection” as follows.

“In order to ensure that the analyzed videos included all the desired elements such as “pediatric consultations”, “multi-party”, and “multilingual interpreter”, three criteria were set for the video selection from the corpus: (1) The age of patient was under 18 years old (pediatric dental consolations); (2) patient was accompanied by a parent/ caregiver (multi-party); and (3) there were Cantonese speaking children and their caregiver/ parent, and a clinical team dyad of non-local clinician (English language-in-use) with the same DSA as the interpreter (multilingual interpreter). Only the recorded videos that fulfilled all the criteria were transcribed and further analyzed in the study.”

**Comments from the Reviewers:**

1. **Is the manuscript technically sound, and do the data support the conclusions? The manuscript must describe a technically sound piece of scientific research with data that supports the conclusions. Experiments must have been conducted rigorously, with appropriate controls, replication, and sample sizes. The conclusions must be drawn appropriately based on the data presented.**

Response: Conversation analysis is a qualitative research method of observation to gather non-numerical data. The conclusions of the study founded on the recurrence patterns identified by the visual text analysis (VTA) and the conversation analysis of the turn-taking in different communication strategies in depth. Also, the rigorous selection of the video ensured that the study analysis aimed at the desired elements such as“pediatric consultations”, “multi-party”, and “multilingual interpreter”. Moreover, Jeffersonian transcription notation was employed to go into detail about the content of conversation and made sure that the replication of the transcript can be obtained by different researchers. Apart from the conversation presentation in text form, the plots obtained from the VTA tools helped to distinguish the different communication strategies. Hence, the conclusion was based on the presented non-numerical data (graphic recurrence pattern and video transcription).

2. **Has the statistical analysis been performed appropriately and rigorously?**

Response: As mentioned, conversation analysis is a qualitative research method. Statistical analysis is not applicable for the non-numerical data in this study.

3. **Have the authors made all data underlying the findings in their manuscript fully available? The PLOS Data policy requires authors to make all data underlying the findings described in their manuscript fully available without restriction, with rare exception (please refer to the Data Availability Statement in the manuscript PDF file). The data should be provided as part of the manuscript or its supporting information, or deposited to a public repository. For example, in addition to summary statistics, the data points behind means, medians and variance measures should be available. If there are restrictions on publicly sharing data—e.g. participant privacy or use of data from a third party—those must be specified.**

Response: Jeffersonian notation for the transcription of the recorded video was attached in S1 File as supporting information. The transcripts of the identified conversations in the study were also attached in S2 File as supporting information. The additional URL link of the VTA software was linked directly from the manuscript file as follows:

“https://www.itee.uq.edu.au/research/projects/discursis”

4. **Is the manuscript presented in an intelligible fashion and written in standard English? PLOS ONE does not copyedit accepted manuscripts, so the language in submitted articles must be clear, correct, and unambiguous. Any typographical or grammatical errors should be corrected at revision, so please note any specific errors here.**

Response: The quality of language has been improved and grammatical errors have been corrected by SMB, CPM and OAZ who are native speakers and/or experts in Literacy. The changes are highlighted in red in the revised manuscript.

**Comments from Reviewer #1:**

1. **I have attached a full review of the paper which is in the main well written. Statistical analysis is not used and does not need to be. I think more information re data collection is required. Please see full review.**

**General: This is an interesting topic and it is good to see Discursis being applied to dentistry. The nature of the study, using children with their parents is also novel. I really like this paper and think it will be a valuable contribution to the literature but, as**

**my comments below suggest, another revision is needed.**

**Introduction: The introduction was appropriate and well referenced. It set the scene with clear research questions that were relevant to the study. There were some awkward turns of phrase that need to be addressed.**

Response: We thank the Review for the advice. We have added the subheading (Multi-party in pediatric dentistry, Multilingual interpreter, Communication strategies, Novel mixed-method approach) into the introduction to provide an organized presentation.

2. **Method: Again, the method section is clear. I think it might be useful to place the communication strategies section in the introduction as it builds on the information provided and does not relate to any methodology and data collection.**

Response: We have moved the “Communication strategies” to the Introduction.

3. **Method: I also think participant information should be in the Method section. In addition, I did not see a full procedure section beyond the little section in 2.1. I really want to see more information that outlines how the study was conducted with respect to recruitment and ages of children, education levels of parents etc. More is required here in order to better understand results.**

Response: The participation information such as the language-in-use of the clinicians, patients, and parent/ primary caregivers, the age of patients, the gender of parents and parent/ primary caregivers were reported in the sub-section “Statistical description of data” in the Result section. However, the education level of parents was not recorded as it was not the focus of this study. Moreover, the sub-section “Data collection” (section 2.1) was extended to include the full study procedure to outline how the study was conducted as follows.

“The dental team and pediatric patients/ primary caregiver (parent) dyads provided written informed consent to video recording of clinical consultations. The information of language-in-use, gender (patients and parent/ primary caregivers only), and age (patients only) was provided by dental team and/or parent/ primary caregivers via a self-complete questionnaire before video recording. In order to ensure that the analyzed videos included all the desired elements such as “pediatric consultations”, “multi-party”, and “multilingual interpreter”, three criteria were set for the video selection from the corpus: (1) The age of patient was under 18 years old (pediatric dental consolations); (2) patient was accompanied by a parent/ caregiver (multi-party); and (3) there were Cantonese speaking children and their caregiver/ parent, and a clinical team dyad of non-local clinician (English language-in-use) with the same DSA as the interpreter (multilingual interpreter). Only the recorded videos that fulfilled all the criteria were transcribed and further analyzed in the study.

The recorded multilingual (Cantonese-English) conversations were transcribed using Transana™ software version 2.53 with Jeffersonian notation (see S1 File). All Chinese characters were parsed into English. Transcriptions and translations were verified by the team members. Data analysis of the transcribed consultations consisted of two phases.

Phase I: Visual text analysis (VTA)….

Phase II: Conversation analysis (CA)….”

4. **Results: If the results section commences at 3.2, then I think more information is required about the three principle dimensions and how they were derived. I am unclear how you decided on the actual plots.**

Response: The three principle dimensions for pattern description of Discursis™ plots were defined by the Discursis’s developers [35]. The detail of the dimensions was graphically presented in the upper right corner Fig 1. We have added more explanations as follows.

“Time scale, direction and interaction type were the three principle dimensions used to define the multi-participant metrics of the recurrence patterns [35]. These indicated whether the recurrences were self/same speaker or other-recurrence, either following a turn (forward-direction) or recurrences related to previous turns (backward-direction) within short, medium or long period of time.

35.	Angus D, Smith A, Wiles J. Human communication as coupled time series: Quantifying multi-participant recurrence. IEEE Trans Audio, Speech, Language Process. 2012;20(6):1795-807.”

5. **Given we know nothing about the participants, it does not assist in understanding the generalizability of these findings.**

Response: We have added the topics of the excerpts and the relevant patient information in the S2 File and the figure legends of Figs 2-7 as follows.

“**Fig 2. Inspect plot of turns 73-81 in Consultation 3 indicating patient-centered direct interpreting.** “The patient’s tooth brushing behaviors” - an eight-year-old girl is here for follow-up and a possible accident causing mal-alignment of teeth.

**Fig 3. Inspect plot of turns 130-145 in Consultation 3 indicating patient-centered mediated interpreting.** “Suggestion for daily tooth brushing” - an eight-year-old girl is here for follow-up and a possible accident causing mal-alignment of teeth.

**Fig 4. Inspect plot of turns 215-227 in Consultation 1 indicating patient-centered direct and mediated interpreting.** “Dental caries prevention work after meals”- an eight-year-old boy previously treated under general anesthesia has erupting permanent teeth.

**Fig 5. Inspect plot of turns 34-66 in Consultation 11 indicating clinician-centered direct interpreting.** “Inquiring about the patient’s daily tooth brushing behaviors” - a seven-year-old boy is here for his six-month review after previous treatment under general anesthesia.

**Fig 6. Inspect plots of turns 18-31 in Consultation 2 indicating clinician-centered mediated interpreting.** “Giving instructions for the pulpectomy treatment” - a seven-year-old boy has missing permanent teeth.

**Fig 7. Inspect plot of turns 127-146 in Consultation 6 indicating clinician-centered direct and mediated interpreting.** “Giving general suggestions to the patient” – a seven-year-old-girl is brought in as she has caries.”

6. **Results: It is always difficult to explain Discursis and the meaning of the diagonals in terms of engagement and time. Too much makes for a complex and unwieldy explanation. Too little leaves the reader unclear. The secret is to strike a balance. I do no think you are there yet. Some earlier explanation about how** **Discursis works would assist. At the moment I think you assume too much knowledge on the part of the reader. As someone who can understand these plots, I understand what you are saying but with the lack of information about participants and the lack of detail on Discursis, I think I would be in the minority. As an example, I believe you need to introduce the idea of recurrence patterns before the Results section (Line 182).**

Response: We have moved the section “Discursis™ plots” (section 3.2) from Result (section 3) to Materials and methods (section 2), and added more explanations about how Discursis works as follows.

“**Discursis™ plots**

Initial, ‘long time scale plots’ as overall conceptual recurrence plots were generated to provide a global overview of both the structure and content of each consultation. Fig 1 illustrates the long-time scale recurrence pattern of a Discursis™ output for Consultation 1. Common topics such as “tooth” and “mouth” that are introduced in the initial phase are repeated and linked across the entire consultation. Time scale, direction and interaction type were the three principle dimensions used to define the multi-participant metrics of the recurrence patterns [35]. These indicated whether the recurrences were self/same speaker or other-recurrence, either following a turn (forward-direction) or recurrences related to previous turns (backward-direction) within short, medium or long period of time. Participation patterns are visualized from the color distributions and the patterns of conceptual recurrence plotting with the color, and the size of boxes in the plots corresponding to the speaker and volume per turn. The color of the lower right corner of the ‘off-diagonal’ boxes represents the speaker of the turn, while the color of the upper left corner of the off-diagonal boxes represents the speaker who shares the repeated topics or concepts. Therefore, a single or a two-colored off-diagonal box refers to the situation where a speaker repeats his or her own topics, or a speaker repeats the topics or concepts from another speaker respectively. Discursis™ inspect plots (for example, see Figs 2-7) provide the zoom-in views of the medium-time recurrence patterns from the overall plots.

…. It is recommended that the plots were read together with the relevant text transcripts in the supporting information to have a better understanding of the results.”

7. **Discussion: I returned to lines 106 to 113 and felt that these aims had not been clearly linked with the results and subsequent discussion. You talk about ‘patient participation’ but remember it is parent participation in this instance.**

Response: We have clearly defined the term “clinician-patient communication” at where it first mentioned in the manuscript as follows.

“… clinician-patient communication (dentists and DSAs vs child patients and their parents/ primary caregivers in paediatric dental practices).”

8. **Discussion: The importance of the mixed methodology was not emphasized sufficiently. Can the use of CA and Discursis be expanded?**

Response: We have added more explanations about the use of CA and Discursis in the sub-section “Principal results” of Discussion as follows.

“The participation patterns were successfully visualized by the recurrence patterns (Multi-colored or monochromatic triangular cluster) in the Discursis^TM^ plots. The preliminary identification of communication styles (PC and CC) can thus be achieved. The in-depth CA of the turns-at-talk among the multiparty participants (dentists and DSAs vs child patients and their parents/ primary caregivers) gave the knowledge of how they construct conversations in the different styles.”

9. **Minor**
   a. **Line 83: Hong Kong is an Asia's world city with a linguistic (should be Asian)**

Response: We thank the Reviewer for the advice of the minor grammatical errors. Brand Hong Kong was launched in 2001 as a government programme designed to promote Hong Kong as "Asia’s world city" (<https://www.brandhk.gov.hk/html/en/>). We have added double quotes to this term.

b. **Line 88: Multilingual interpreters are essential resource (needs ‘an’)**

Response: We have added the word “an” in line 88 as follows.

“Multilingual interpreters are an essential resource in between English-speaking clinicians and the Cantonese-speaking patients for the multilingualism in the dentistry in Asia [13].”

c. **Line 94: …. Challenges are existed (needs changing)**

Response: We revised the sentence in line 94 as follows.

“However, there are challenges in the medical information extraction from the clinician-patient communication.”

d. **Line 110: The study also targeted to provide (needs changing)**

Response: We revised the sentence in line 110 as follows.

“The study also aimed at providing an evidentiary account of …”

**Comments from Reviewers #2:**

1. **The authors aimed to ‘identify the oral health literacy (OHL) talk in interpreter-mediated paediatric dentistry and (2) to analyze the interpreter contribution to the communication strategies’. They used mixed-methods combining VTA and CA to investigate a large number of video recorded consultations in paediatric dental practices. However, the analyses seem rather underdeveloped, and the presentation is crippled by poor quality language and structure.**

Response: We thank the Reviewers for the comments on the manuscript. Conversation analysis is a qualitative research method of observation to gather non-numerical data. Therefore, statistical analysis is not applicable for the non-numerical data in this study. The quality of language has been improved and grammatical errors have been corrected by SMB, CPM and OAZ who are native speakers and/or experts in Literacy. The changes are highlighted in red in the revised manuscript. The presentation structure has been revised as follows.

“Introduction

-Multi-party in pediatric dentistry

-Multilingual interpreter

-Communication strategies

-Novel mixed-method approach

Materials and methods

-Data collection

-Phase I: Visual text analysis (VTA)

-Discursis™ plots

-Phase II: Conversation analysis (CA)

Results

-Statistical description of data

-Patient-centered (PC) communication

-Patient-centered direct interpreting (PC-DI)

-Patient-centered mediated interpreting (PC-MI)

-Patient-centered direct interpreting and mediated interpreting (PC-DI and MI)

-Clinician-centered (CC) communication

-Clinician-centered direct interpreting (CC-DI)

-Clinician-centered mediated interpreting (CC-MI)

-Clinician-centered direct interpreting and mediated interpreting (CC-DI and MI)

-Discussion

-Comparison with prior work

-Principal results

-Limitations

-Next steps

-Conclusions”

2. **The literature review has not sufficiently established the knowledge gaps. Although there is little literature on interpreter use in dentistry and paediatrics (but not non-existing), how the studies in other fields can inform the understanding of the situations in hand?**

Response: The key concept be reviewed in the 2^nd^ paragraph of Introduction was “Multilingual interpreter”. Therefore, we added the subheading “Multilingual interpreter” to clarify the section. We first reviewed studies in dental [14] and medical consultations [15]. We then reviewed studies in other fields [16-20]. Finally, the need of “Multilingual interpreter” in the pediatric dentistry in Hong Kong was brought out to explain the knowledge gap [21,22].

14.	Bridges S, Drew P, Zayts O, McGrath C, Yiu CKY, Wong HM, et al. Interpreter-mediated dentistry. Soc Sci Med. 2015;132:197-207.

15.	Bolden GB. Toward understanding practices of medical interpreting: Interpreters' involvement in history taking. Discourse Stud. 2000;2(4):387-419.

16.	Mason I. On mutual accessibility of contextual assumptions in dialogue interpreting. J Pragmat. 2006;38(3):359-73.

17.	Baker M. Contextualization in translator-and interpreter-mediated events. J Pragmat. 2006;38(3):321-37.

18.	Setton R. Context in simultaneous interpretation. J Pragmat. 2006;38(3):374-89.

19.	Johnstone B. Place, globalization, and linguistic variation 2004. 65-83 p.

20.	Li DC. Cantonese‐English code‐switching research in Hong Kong: A Y2K review. World Englishes. 2000;19(3):305-22.

21.	Meyer B, Bührig K. Interpreting risks. Medical complications in interpreter-mediated doctor-patient communication. European J of Appl Linguist. 2014;2(2):233-53.

22.	Brunner J, Chuang E, Goldzweig C, Cain CL, Sugar C, Yano EM. User-centered design to improve clinical decision support in primary care. Int J Med Inform. 2017;104:56-64.

3. **The authors mention several key concepts, such as patient-centred communication, but they are insufficiently explained. It seems the authors were analysing how patient-centred (PC) approaches were used in OHL talks but it is unclear what and how established PC models were use in the analysis.**

Response: We have moved the “Communication strategies” from the section “Materials and method” to “Introduction”. This subsection explains how the patient-centered communication is qualitatively defined. More explanations have been added to “Materials and method” and the subsection of “Patient-centered (PC) communication” of “Results”, which would help readers understand what and how the established PC models were used in the analysis.

4. **The methods section does not give sufficient detail of the CA and VTA and how VTA informed CA. When doing VTA, what model was used to identify relevant analytic moments? More detail is required to allow better understanding of how the two methods intersected in the analysis.**

Response: We have moved the section “Discursis™ plots” (section 3.2) from Result (section 3) to Materials and methods (section 2), and added more explanations about how VTA (Discursis) works and how it intersected with CA as follows.

“Discursis™ plots

Initial, ‘long time scale plots’ as overall conceptual recurrence plots were generated to provide a global overview of both the structure and content of each consultation. Fig 1 illustrates the long-time scale recurrence pattern of a Discursis™ output for Consultation 1. Common topics such as “tooth” and “mouth” that are introduced in the initial phase are repeated and linked across the entire consultation. Time scale, direction and interaction type were the three principle dimensions used to define the multi-participant metrics of the recurrence patterns [35]. These indicated whether the recurrences were self/same speaker or other-recurrence, either following a turn (forward-direction) or recurrences related to previous turns (backward-direction) within short, medium or long period of time. Participation patterns are visualized from the color distributions and the patterns of conceptual recurrence plotting with the color, and the size of boxes in the plots corresponding to the speaker and volume per turn. The color of the lower right corner of the ‘off-diagonal’ boxes represents the speaker of the turn, while the color of the upper left corner of the off-diagonal boxes represents the speaker who shares the repeated topics or concepts. Therefore, a single or a two-colored off-diagonal box refers to the situation where a speaker repeats his or her own topics, or a speaker repeats the topics or concepts from another speaker respectively. Discursis™ inspect plots (for example, see Figs 2-7) provide the zoom-in views of the medium-time recurrence patterns from the overall plots. The qualitative analysis of the turns-at-talk among the multiparty participants was conducted in Phase II using CA.”

5. **The results section is most under developed. It is generally descriptive and anecdotal. The presentation fails to demonstrate trend which is expected of VTA or to provide rich description of turn by turn interaction, which is expected of CA. At times the presentation was so descriptive that it lost the purpose of the overarching aim.**

Response: Descriptive and anecdotal are characteristics for results of Qualitative research (Mayes et al 2007). We have added more explanations to emphasize the importance of CA and Discursis in this study in the sub-section “Principal results” of Discussion as follows.

“The participation patterns were successfully visualized by the recurrence patterns (Multi-colored or monochromatic triangular cluster) in the Discursis^TM^ plots. The preliminary identification of communication styles (PC and CC) can thus be achieved. The in-depth CA of the turns-at-talk among the multiparty participants (dentists and DSAs vs child patients and their parents/ primary caregivers) gave the knowledge of how they construct conversations in different styles.”

Mayes LE, Fonagy PE, Target ME. Developmental science and psychoanalysis: Integration and innovation: Karnac Books; 2007.

6. **The simple subheadings, such as PC-DI, do not provide sufficient explanation of data nor do they contribute new knowledge.**

Response: We have revised the subheadings as follows.

“-Patient-centered (PC) communication

-Patient-centered direct interpreting (PC-DI)

-Patient-centered mediated interpreting (PC-MI)

-Patient-centered direct interpreting and mediated interpreting (PC-DI and MI)

-Clinician-centered (CC) communication

-Clinician-centered direct interpreting (CC-DI)

-Clinician-centered mediated interpreting (CC-MI)

-Clinician-centered direct interpreting and mediated interpreting (CC-DI and MI)”

7. **The authors have collected very rich data. The mixed methods approach has great potential to provide complex interpretation of the interactions in dental paediatrics. With more in-depth analysis this research may make valuable contribution to the literature on interpreter mediated consultations. Better presentation with improved language can help convey the message better.**

Response: Your comments are very much appreciated. Not many readers are familiar with Discursis, so much space of this paper is devoted to help the readers understand the results. Concerning the focus and length of this paper, more analyses will be conducted in the future.

We hope you are satisfied with our responses.

Best regards,

Dr. Hai Ming Wong

Clinical Associate Professor

Department of Paediatric Dentistry, Faculty of Dentistry, The University of Hong Kong

Address: PDO, 2/F Prince Philip Dental Hospital, 34 Hospital Road, HK

Fax: 25593803

Tel: 28590261

Email: [wonghmg@hku.hk](mailto:wonghmg@hku.hk)
