# Supplementary material for: Advanced informatics understanding of clinician-patient communication: A mixed-method approach to oral health literacy talk in interpreter-mediated pediatric dentistry
Source: PLoS One. 2020 Mar 20;15(3):e0230575. doi: 10.1371/journal.pone.0230575 (PMC7083275; doi:10.1371/journal.pone.0230575)
Supplement: Attachment — Submitted filename: PONE-D-19-27918R.docx [file pone.0230575.s005.docx]

PONE-D-19-27918R

Revision

I congratulate the authors on the revisions. I think the paper is much clearer than before and is a valuable contribution to the literature.

I think there may be a typo on line 262 where the authors say, “The greed-blue and pink-blue……”. Should be this green-blue?

I have one further request which may reflect my discipline, but I would like to see the aims explicitly revisited in the Discussion. In particular the second aim concerning PC-DI, PC-MI, CC-DI and CC-MI.

They are clearly described in the results but then not further discussed later. I think you could do more in the discussion to clarify and round off the paper.
