# Supplementary material for: Advanced informatics understanding of clinician-patient communication: A mixed-method approach to oral health literacy talk in interpreter-mediated pediatric dentistry
Source: PLoS One. 2020 Mar 20;15(3):e0230575. doi: 10.1371/journal.pone.0230575 (PMC7083275; doi:10.1371/journal.pone.0230575)
Supplement: Attachment — Submitted filename: Response to Reviewers_R2.docx [file pone.0230575.s006.docx]

Dear Prof Barbara Schouten, Academic Editor,

Thank you for considering our research article entitled “Advanced informatics understanding of clinician-patient communication: a mixed-method approach to oral health literacy talks in interpreter-mediated pediatric dentistry” (PONE-D-19-27918R1). Enclosed please find a marked-up copy of our manuscript that highlights changes, and an unmarked version of our revised paper without tracked changes. With regard to the Academic Editor and Reviewers’ comments, we provide the following point by point response and explanation.

**Comments from the Academic Editor:**

1. **Both reviewers were in general satisfied with your revision. However, they have some minor comments that should be addressed; in particular, as suggested by reviewer 1, the discussion-section can be enhanced a bit.**

Response: We thank the reviewers’ agreements on the previous revisions. We have revised the manuscript to meet the reviewers’ new comments.

2. **In addition, I would add the fact that you did not assess parent's educational level as a limitation of your study, because there is a clear link established in previous research between educational level on the one hand, and health literacy and patient participation on the other hand. Please briefly address this as well in your discussion section.**

Response: We thank the Editor for the concern about the effect of educational level of the patients in the study. We have added the discussion on the study limitations and the study contribution to future work in the sub-sections “Discussion – Limitations” and “Discussion – Next steps” as follows.

“The main focuses of the study were to investigate the recurrence patterns of OHL talks in interpreter-mediated pediatric dentistry and to qualitatively explore the content of the talks. “Language-in-use” of the multi-parties was the major criterion in the selection of the OHL talks including a multilingual interpreter. Despite the randomness of patient background, the same DAS (multilingual interpreter) was involved in all the selected videos to reduce the effects of clinicians’ background. However, the effects of parents and patients’ socio-economic status [54] such as educational level on communication strategies, or the effects of communication strategies on the outcomes [3, 4] such as their satisfaction, were not investigated and discussed in this study. Those effects need to be further quantitatively investigated for designing a guideline on good health communication practices.”

54.	Willems S, De Maesschalck S, Deveugele M, Derese A, De Maeseneer J. Socio-conomic status of the patient and doctor–patient communication: does it make a difference? Patient Educ Couns. 2005;56(2):139-46.

“In an ideal situation, a better clinician-patient communication can be achieved by all multi-parties with higher educational levels. Although the educational levels of patients or their parents are not controllable, the communication skills of dental professionals are educable. The popularization of the use of VTA……”

**Comments from Reviewer #1:**

1. **I congratulate the authors on the revisions. I think the paper is much clearer than before and is a valuable contribution to the literature.**

Response: We thank the Review for the agreement on the revision and the contribution of the paper.

2. **I think there may be a typo on line 262 where the authors say, “The greed-blue and pink-blue……”. Should be this green-blue?**

Response: We have revised the sentence in line 262 as follows.

“The green-blue and pink-blue ……”

3. **I have one further request which may reflect my discipline, but I would like to see the aims explicitly revisited in the Discussion.** **In particular the second aim concerning PC-DI, PC-MI, CC-DI and CC-MI. They are clearly described in the results but then not further discussed later. I think you could do more in the discussion to clarify and round off the paper.**

Response: We have explicitly revisited the two research questions (aims) of the study in the sub-sections “Discussion – Principal results” to clarity and round off the paper as follows.

“With regard to the *1^st^* research question ‘*where*’ were the OHL talks, the clinician-dominated and multi-party participation patterns were successfully visualized by the recurrence patterns of monochromatic and multi-colored triangular cluster in the Discursis™ plots, respectively. The former pattern was preliminarily identified to be CC communication style, while the latter was PC communication style. Conventionally, it was hard to quantitatively assess the level of interactive engagement of participants in conversations. This increased the difficulties in identifying different communication styles. The study results showed that the application of the VTA can be an alternative to the preliminary identification of the communication styles, and a valuable approach of the PC communication studies. Subsequently, the in-depth CA of the turns-at-talk among the multiparty participants (dentists and DSAs vs child patients and their parents/ primary caregivers) gave the knowledge of ‘*how*’ multi-party, multilingual pediatric dental consultations were interactively managed – the *2^nd^* research question. The two communication styles (PC and CC) can then be further divided in to three strategies (-DI, -MI, and -DI and MI). DI illustrated how the DSA (multilingual interpreter) cooperated with dentists, and MI illustrated how the interpreter tried to build a connection with the patients/ primary caregivers. The mix of DI and MI further showed how the multi-party, multilingual pediatric dental consultations were constructed by the interactions of the participants. The importance of multilingual interpreter was demonstrated in both unveiled communication strategies.”

**Comments from Reviewers #2:**

1. **The revision has improved significantly. The language flows much smoother and is easy to read. The details about the two-phased analysis provide clarity on how the mixed methods approach was implemented in this study, which was one of my questions.**

Response: We thank the Review for the agreement on the revisions and the study approach.

2. **I feel the authors do not need to refer to patient-centred communication in this study. I find it problematic to determine whether the interaction is patient-centred purely based on the level of engagement of the parent and the patient in the interaction. It is well established that patients speak less in information giving/explaining stage of a consultation, and this does not mean the clinician has taken a non-patient-centred approach. This is true in the authors' study. I read the transcript, where I can see a lot of good practices of the clinicians that would be deemed as patient centred. For example, they adapted the language when giving instructions to make it simple and digestible for children. The focus of this study is to** **unveil the patterns. This is already achieved without referencing patient-centred communication.**

Response: Since the introduction of the PC communication is important to differentiate between the two unveiled recurrence patterns (monochromatic and multi-colored triangular cluster) in the Discursis™ plots of the study, we need to keep the refereeing process. In addition, one of the difficulties in identifying communication styles is that communication styles cannot be easily quantitatively defined. It is the reason why visual text analysis (VTC) was needed in this study and how this study contributed to studies of PC communication. We have added more explanations about importance of the study results to the field of PC communication in the sub-sections “Discussion – Principal results” as follows.

“With regard to the *1^st^* research question ‘*where*’ were the OHL talks, the clinician-dominated and multi-party participation patterns were successfully visualized by the recurrence patterns of monochromatic and multi-colored triangular cluster in the Discursis™ plots, respectively. The former pattern was preliminarily identified to be CC communication style, while the latter was PC communication style. Conventionally, it was hard to quantitatively assess the level of interactive engagement of participants in conversations. This increased the difficulties in identifying different communication styles. The study results showed that the application of the VTA can be an alternative to the preliminary identification of the communication styles, and a valuable approach of the PC communication studies.”

3. **I think the transcript can be improved. The authors claim that they used Jeffersonian conventions; however, the presentation of the transcripts are not strictly following the conventions. For example, pauses/gaps are usually marked like e.g. (0.96), with the number inside to indicate the length of a pause.**

Response: In Jeffersonian intonation marks, the time of the pauses is accurate to 0.5s ((.): noticeable pause shorter than 0.5 s; (~n): timed pause in n integer second). We have synchronized the intonations (e.g. (short pause), (pause), (SP), and (0.1)) to the required intonation (.) in the revised S2 File. The revise S2 file is also attached for the reference.

4. **Overlapping speeches should be aligned with each other marked with [.**

Response: The focus of the study was the text content of the conversation. Also, the bilingual transcripts will become messy and plenty of empty spaces will be generated, if the content of the conversation is forced to be aligned. We therefore decided not to align the text of the overlapping speeches in the transcripts.

5. **There are still some typos in the manuscript. Here are some that I have noticed:**
a. **I don’t understand the sentence in lines 90-92. Maybe rephrase.**

Response: We have rephrased the sentence in lines 90-92 as follows.

“Research in ad hoc interpreting has raised concerns regarding the communication of the risks in medical complications [21] and the improvements in clinical shared decision-making [22].”

b. **98—healthcare communication not health sciences communication**

Response: We have revised the word “healthcare” in line 98 as follows.

“……classifications in healthcare communication.”

c. **137—what’s the average length of consultation? What’s the total hours of recording?**

Response: The average length and the total hours of the larger video-recorded corpus (n=199), including the videos not fulfilling the selection criteria, were irrelevant and were excluded from the study analysis. The average length (12.62 mins) and total hours (12.62*77 = 971.74 mins or 16.2 hours) of the selected videos have been already presented in lines 198-199 as follows.

“The average length and turns-at-talk of the 77 transcribed video recordings were 12.62 minutes and 290 turns, respectively.”

d. **189—may be delete ‘with turns-at-talk overlap’.**

Response: We have deleted the words “with turns-at-talk overlap” in line 189.

e. **193—Spell out MI.**

Response: The short form of the term “mediated interpreting (MI)” was first mentioned in line 84 of the paper. We therefore keep the use of the short form MI in line 193.

We hope you are satisfied with our responses.

Best regards,

Dr. Hai Ming Wong

Clinical Associate Professor

Department of Paediatric Dentistry, Faculty of Dentistry, The University of Hong Kong

Address: PDO, 2/F Prince Philip Dental Hospital, 34 Hospital Road, HK

Fax: 25593803

Tel: 28590261

Email: [wonghmg@hku.hk](mailto:wonghmg@hku.hk)
